# Interplay of virulence factors and signaling molecules: albumin and calcium-mediated biofilm regulation in *Bordetella bronchiseptica*

**DOI:** 10.1128/jb.00445-24

**Published:** 2025-03-26

**Authors:** Sabrina Laura Mugni, Nicolás Ambrosis, George A. O´Toole, Federico Sisti, Julieta Fernández

**Affiliations:** 1 CCT La Plata. CONICET. Departamento de Ciencias Biológicas, Facultad de Ciencias Exactas, UNLP, Instituto de Biotecnología y Biología Molecular 124456 , La Plata, Buenos Aires Province, Argentina; 2 Department of Microbiology and Immunology, Geisel School of Medicine at Dartmouth 12285, Hanover, New Hampshire, USA; NCBI, NLM, National Institutes of Health, Bethesda, Maryland, USA

**Keywords:** biofilms, *Bordetella*, c-di-GMP, host factors

## Abstract

*Bordetella bronchiseptica,* a respiratory pathogen capable of infecting various mammals, including humans, is associated with chronic infections. *B. bronchiseptica* can form biofilm-like structures *in vivo*, providing tolerance against environmental stresses. Recent studies have highlighted the role of cyclic diguanylate monophosphate (c-di-GMP) in this process *in vitro*: elevated c-di-GMP levels stimulate biofilm formation, whereas phosphodiesterase (PDE) activation reduces biofilms. Respiratory secretions, which contain albumin and calcium at higher concentrations than standard growth media, promote an increase in the amount and extracellular localization of the adenylate cyclase toxin (ACT), an important virulence factor of *Bordetella spp*. Secreted ACT, present in the extracellular medium or attached to the outer membrane, inhibits biofilm formation. Based on these observations, we hypothesized that serum albumin and calcium together inhibit biofilm formation and explored the potential role of c-di-GMP in this process. Our findings suggest that serum albumin and calcium inhibit *B. bronchiseptica* biofilm formation through two potentially independent mechanisms: one involving ACT secretion and another promoting c-di-GMP degradation. In the presence of albumin and calcium, intracellular levels of c-di-GMP were reduced, and specific PDEs appear to be involved in this process. In addition, albumin and calcium stimulated the secretion of the adhesin BrtA. This study contributes to the understanding of the mechanisms governing *B. bronchiseptica* biofilm formation and its modulation by host factors.

**IMPORTANCE:**

*Bordetella bronchiseptica*, a respiratory pathogen capable of infecting various mammals, forms biofilms that enhance its ability to withstand environmental stresses. This study reveals that host-derived factors, specifically serum albumin and calcium, inhibit biofilm formation through two independent mechanisms: increasing adenylate cyclase toxin secretion and promoting the degradation of cyclic diguanylate monophosphate (c-di-GMP), a key biofilm regulator. These findings provide insights into how host conditions influence *B. bronchiseptica* biofilm dynamics, shedding light on the complex interactions between pathogen and host that contribute to infection persistence. Understanding these mechanisms may inform strategies to mitigate chronic infections caused by *B. bronchiseptica*.

## INTRODUCTION

*Bordetella bronchiseptica* is a respiratory pathogen known to infect a variety of mammals, including humans. Infections caused by this pathogen are characterized by chronic and persistent infections, contrasting with the more acute infections caused by the human-exclusive pathogen *B. pertussis*. It is increasingly accepted that both pathogens can survive asymptomatically in the upper respiratory tract for extended periods, such as up to 30 days post-infection (dpi) for *B. pertussis* in mice, and 56 dpi for in swine and 45 dpi in mice for *B. bronchiseptica* (1–3). This asymptomatic persistence may explain the difficulty in eradicating *B. pertussis* despite high vaccine coverage.

Biofilm formation by *B. bronchiseptica* is a highly regulated process controlled in part by the two-component system BvgAS, which also regulates the expression of virulence factors (4). While the BvgAS system is hypothesized to be constitutively active, its activity can be modulated in the laboratory by millimolar concentrations of nicotinic acid or magnesium sulfate. Intermediate concentrations of these modulators can induce an “intermediate phase” in *B. bronchiseptica* where several virulence factors are absent, yet biofilm formation is maximized (4). Recent investigations by our group have highlighted the role of the second messenger cyclic diguanylate monophosphate (c-di-GMP) in regulating biofilm formation by *B. bronchiseptica*, particularly during the intermediate phase (5).

Elevated levels of c-di-GMP, resulting from the activation of diguanylate cyclases (DGC), stimulate biofilm formation, whereas activation of phosphodiesterases (PDEs) leads to c-di-GMP degradation and subsequent biofilm reduction. The BrtA/Lap system, responsive to c-di-GMP levels, is required for biofilm formation in the intermediate phase (6). BrtA, a large adhesin, remains attached to the outer membrane until its cognate protease, LapG, cleaves the N-terminal periplasmic domain of this adhesin. The release of BrtA is associated with low biofilm levels. LapG, a periplasmic protease, is sequestered by LapD when cytosolic c-di-GMP concentrations are high, preventing BrtA cleavage. Hence, if c-di-GMP is elevated, BrtA remains on the bacterial surface, and biofilm is formed and stabilized.

Both *B. pertussis* and *B. bronchiseptica* can form biofilm-like structures *in vivo* on the respiratory epithelium (7, 8). Biofilm formation is typically described as a tolerance mechanism to protect against antibiotics and other environmental stresses. The biofilm matrix of *B. bronchiseptica* is comprised of proteins, polysaccharides, and extracellular DNA (eDNA), which provide structural integrity and protection for the bacterial community (9). Key adhesins such as filamentous hemagglutinin (FHA) play pivotal roles in biofilm formation and can be secreted into the extracellular milieu (10). Interestingly, the presence of adenylate cyclase toxin (ACT) has been found to diminish biofilm formation (11). Deleting the *cyaA* gene encoding the ACT of *B. bronchiseptica* increases biofilm formation, while the addition of the ACT to the extracellular medium inhibits biofilm formation by *Bordetella pertussis* (4, 11).

Respiratory secretions, which contain albumin and calcium, promote an increase in the amount and membrane localization of active ACT (12). This increased secretion and surface localization of ACT can be elicited *in vitro* by both serum and albumin. The response to albumin is not mediated through the regulation of ACT at the transcriptional level or activation of the BvgAS two-component system (12). Considering the promotion of ACT secretion by serum albumin and its reported involvement in biofilm formation, we hypothesized that serum albumin may inhibit biofilm formation due at least in part to the production of ACT. Furthermore, given the regulatory role of c-di-GMP in biofilm formation, we aimed to investigate whether this second messenger is involved in the signaling triggered by serum albumin.

Here we discovered that bovine serum albumin (BSA) and calcium inhibit the formation of *B. bronchiseptica* biofilms. While the FHA adhesin does not appear to be involved in this process, ACT is implicated, with increased ACT levels associated with reduced biofilms. In addition, we show that the surface localization of the BrtA adhesin, responsible for biofilm formation in the intermediate phase, is regulated by BSA and calcium through the activation of PDEs, which, in turn, reduce c-di-GMP levels. Through this study, we seek to deepen our understanding of the mechanisms underlying *B. bronchiseptica* biofilm formation and its modulation by host factors.

## RESULTS

### Albumin inhibits *B. bronchiseptica* biofilm formation

Gonyar and colleagues demonstrated that albumin and calcium present in the host serum were responsible for an increase in ACT secretion into the extracellular media (12). In addition, ACT present in the extracellular media, and possibly on the external surface of the bacteria, inhibits biofilm formation (11). Taking these two results together, we hypothesized that albumin and calcium at serum concentrations would inhibit biofilm formation. To test this hypothesis, we evaluated biofilm formation using the crystal violet (CV) technique in the absence and presence of albumin and calcium. Although not specific to biofilm formation, CV staining has been widely used as a convenient method to detect and quantify biofilm-like structures. Following the methods of Gonyar et al., we used bovine serum albumin (BSA, 2 mg/mL) plus CaCl_2_ 2 mM (12) in our assays.

**Fig 1:**
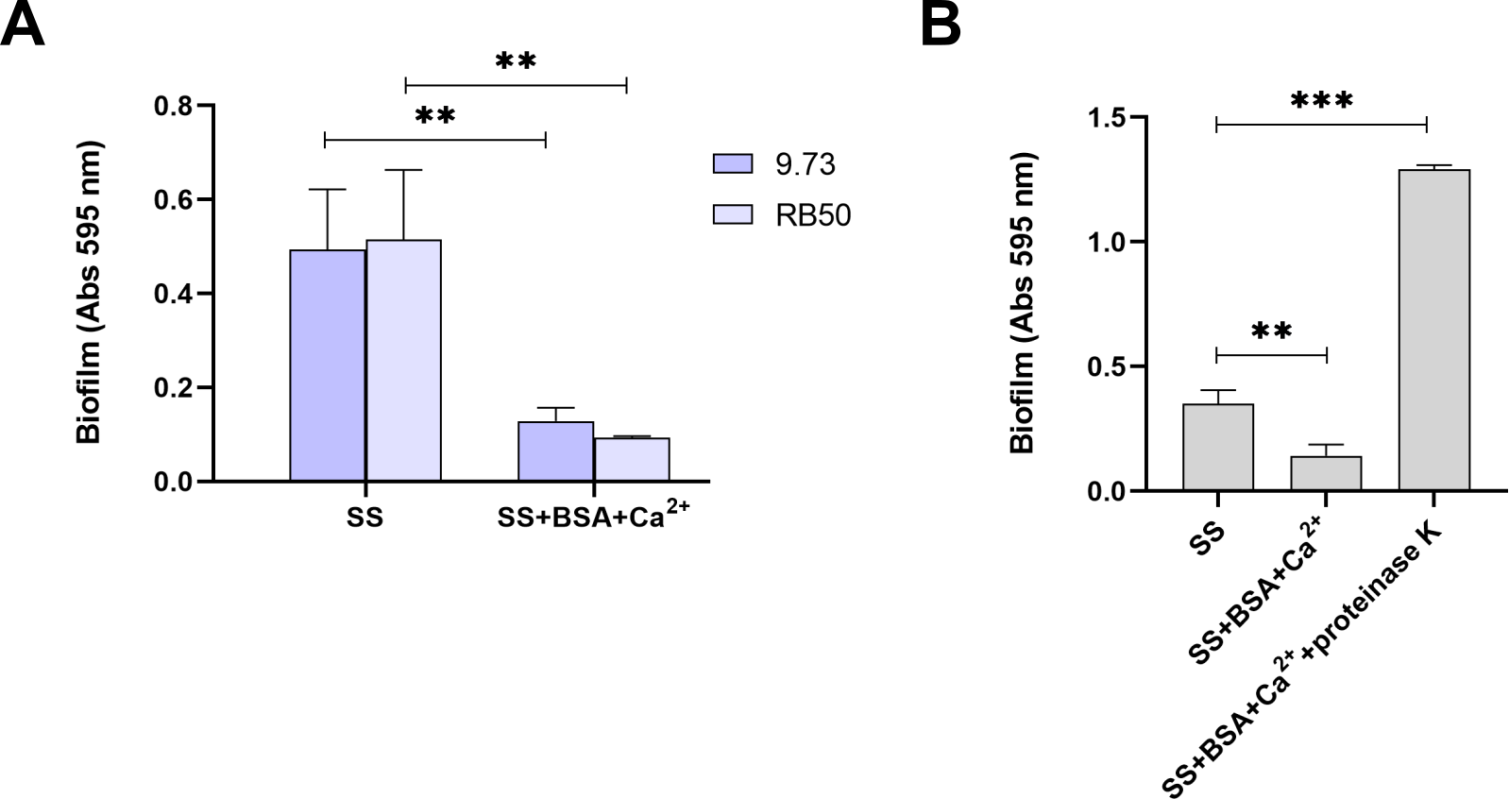
Albumin and calcium inhibit *B. bronchiseptica* biofilm formation. (A) Biofilm formation on PVC 96-well plates was assessed of overnight cultures of wild-type *B. bronchiseptica* 9.73 or RB50 grown in SS or SS supplemented with 2.0 mg/mL BSA and 2.0 mM CaCl_2_. (B) Biofilm formation of *B. bronchiseptica* 9.73 in SS supplemented with BSA+Ca^2+^ pretreated with proteinase K. Visualization and quantification were performed using the CV staining technique. Results are the average of at least three independent experiments. ** indicates significant differences *P* < 0.01 (ANOVA).

As shown in Fig. 1, significant inhibition of biofilm formation was noted in the presence of BSA+Ca^2+^ at serum-relevant concentrations for *B. bronchiseptica* 9.73. The reduction was not attributable to differences in growth. The effect was also observed in *B. bronchiseptica* strain RB50 (Fig. 1A). For the remaining experiments described herein, the 9.73 strain or its derived mutants were used exclusively. Inhibition was not observed when BSA was pretreated with proteinase K, thus ruling out potential effects of contaminants present in the BSA (Fig. 1B).

To test the effects of both signals separately, we evaluated biofilm formation only in the presence of either BSA or calcium at varying concentrations. Interestingly, calcium alone increased biofilm formation, while albumin alone did have a significant effect (). Previous observations by Gonyar et al. (12) reported minimal ACT secretion in the presence of albumin alone but a significantly higher effect when albumin and calcium were combined (12). Based on these findings, we focused on the combined effect of albumin and calcium to better mimic *in vivo* conditions, as previously suggested by Bumba and colleagues (13).

**Fig 2:**
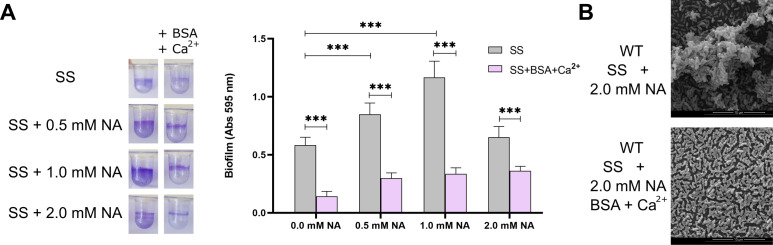
The effect of BSA+Ca^2+^ on biofilm formation is independent of the virulent phase. (A) Wild-type *B. bronchiseptica* 9.73 was grown for 24 hours in SS or SS supplemented with 2.0 mg/mL BSA and 2.0 mM CaCl_2_ with the indicated final concentration of added nicotinic acid (NA). Biofilm was stained with CV and quantified after resuspension in 33% (vol/vol) acetic acid. Results are the average of at least three independent experiments. *** indicates significant differences versus SS at the same nicotinic acid concentration (*P* < 0.001. ANOVA). (B) Scanning electron microscopy of 24 h cultures of wild-type *B. bronchiseptica* grown in SS with 2.0 mM NA or in SS with 2.0 mM NA, 2.0 mg/mL BSA, and 2.0 mM CaCl_2_.

*B. bronchiseptica* biofilm formation is regulated by the two-component system BvgAS. When partially active, meaning in the intermediate phase, *B. bronchiseptica* forms the maximum biofilm. To determine whether BSA+Ca^2+^ inhibition is also observed in other phases besides the virulent phase, we conducted static biofilm assay experiments with bacteria modulated in intermediate and avirulent phases using nicotinic acid (NA). As previously described, biofilm formation was higher in the intermediate phase, that is, in the presence of 0.5–2 mM NA. In all NA concentrations tested, BSA+Ca^2+^ inhibited biofilm formation (Fig. 2A).

Biofilm inhibition in the intermediate phase was confirmed by scanning electron microscopy (SEM) (Fig. 2B). We chose the intermediate phase for SEM observation because it was the condition where some biofilm persisted even in the presence of BSA+Ca^2+^. When *B. bronchiseptica* was grown in SS with 2 mM NA, it formed three-dimensional structures, but in the presence of BSA+Ca^2+^, only a monolayer was observed (Fig. 2B). Taken together, our results confirm our initial hypothesis that host serum components, albumin plus calcium, inhibit *B. bronchiseptica* biofilm formation.

### Filamentous hemagglutinin is not required for the reduction in biofilm formation by added BSA plus calcium

FHA is an adhesin located at the bacterial surface, crucial for biofilm formation. FHA can be cleaved and released into extracellular media by a tightly regulated process involving various proteases, including DegP (14, 15). Serra and co-workers reported inhibition of *B. pertussis* biofilm formation by exogenous addition of FHA (10), suggesting that interfering with FHA-mediated interactions, possibly through the production of proteolyzed FHA, could reduce biofilm formation. We hypothesized that BSA+Ca^2+^ increases FHA secretion, leading to a reduction in biofilm formation. If this hypothesis is correct, a *fhaB* mutant (lacking FHA production) would display reduced biofilm levels in SS, regardless of the addition of BSA+Ca^2+^. This phenotype was previously described by Irie et al. (4), and we confirmed this observation with the *Bb*9.73 strain in our laboratory () (4). Assessing FHA secretion in a *fhaB* mutant is not feasible, as the protein is not produced in this strain. For these reasons, we chose to test our hypothesis using a *degP* mutant, which affects FHA secretion without eliminating its production, allowing us to study the effect of altered FHA secretion on biofilm formation. If our hypothesis is correct, the deletion of *degP*, which is essential for the release of FHA, would reverse the negative effect of BSA+Ca^2+^ on biofilm formation.

**Fig 3:**
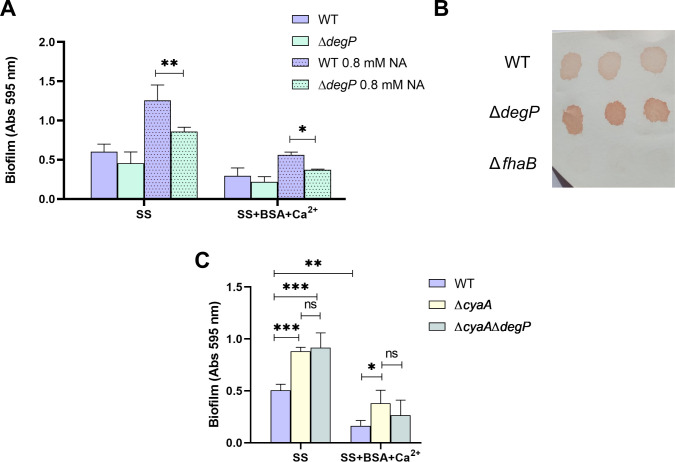
The role of filamentous hemagglutinin and adenylate cyclase toxin on biofilm formation. Biofilm formation on PVC 96-well of overnight cultures of wild-type *B. bronchiseptica* and *Bb*Δ*degP* (**A**) or *Bb*Δ*cyaA* and *Bb*Δ*cyaA*Δ*degP* (**C**) grown in SS or SS supplemented with 2.0 mg/mL BSA and 2.0 mM CaCl_2_. When indicated, NA 0.8 mM final concentration was added. Biofilm was stained with CV and quantified after resuspension in 33% (vol/vol) acetic acid. Results are the average of at least three independent experiments. ** and *** indicate significant differences (*P* < 0.01 and *P* < 0.001, respectively. ANOVA). (B) Cell surface levels of FHA in cells grown in SS 0.8 mM NA as measured by dot blot. A representative dot blot assay with triplicates is shown.

*B. bronchiseptica* with a clean deletion of the *degP* gene was evaluated for biofilm formation. In the absence of the *degP* gene, BSA+Ca^2+^ continued to reduce biofilm formation (Fig. 3A). Given that the effect of FHA on biofilm formation is most evident in SS medium with 0.8 mM NA, we next performed biofilm experiments under these conditions (SS +0.8 mM NA). While the absence of FHA did not alter the reduction in biofilm formed upon the addition of BSA+Ca^2+^ (), the deletion of *degP* significantly impaired biofilm formation in the absence of BSA+Ca^2+^. These data suggest that FHA released from the cell surface likely promotes biofilm formation under these conditions (Fig. 3A), but the biofilm is still reduced by the addition of BSA+Ca^2+^. Differences were not due to an effect on growth ().

Dot blot analysis of intact cells using an anti-FHA antibody revealed a stronger FHA signal in the *Bb*Δ*degP* strain compared to the isogenic parent strain (Fig. 3B), indicating that the accumulation of FHA on the cell surface despite the reduction in biofilm formation upon BSA+Ca^2+^ addition. Together, these data indicate that biofilm inhibition by BSA+Ca^2+^ is not due to a reduction in FHA levels.

### *Adenylate cyclase toxin-mediated biofilm inhibition by BSA+Ca* ^2+^

Adenylate cyclase toxin, via its AC domain, binds FHA on the cell surface, thus preventing FHA interactions necessary for biofilm formation (11). Irie and co-workers reported increased biofilm formation in the absence of ACT (4). As described previously, albumin and calcium stimulate ACT secretion. Considering all these data, we hypothesized that the observed biofilm inhibition in the presence of BSA+Ca^2+^ is caused by increased secreted ACT. To test this idea, we first evaluated biofilm formation by a *cyaA* mutant (*Bb*Δ*cyaA*) to confirm the results obtained by Irie and co-workers (Fig. 3C). The *Bb*Δ*cyaA* mutant formed more biofilm than the wild-type strain in the virulent phase and also formed more biofilm than the WT in the presence of BSA+Ca^2+^ (Fig. 3C).

To rule out the possibility that FHA plays a role in biofilm reduction but that this effect was masked by the ACT effect, we evaluated biofilm formation in a double mutant strain lacking both *degP* and *cyaA*. No significant differences were observed between *Bb*Δ*cyaA*Δ*degP* and *Bb*Δ*cyaA* strains (Fig. 3C). Given that the strain lacking ACT showed less inhibition than the WT, these data indicate that ACT may be involved in biofilm inhibition by added BSA+Ca^2+^.

### BSA+Ca^2+^-mediated decrease in c-di-GMP levels correlates with biofilm reduction

We previously described that c-di-GMP levels regulate biofilm formation by *B. bronchiseptica*, with high c-di-GMP levels being associated with enhanced biofilm (5). If biofilm inhibition mediated by BSA+Ca^2+^ is partially or fully driven by changes in c-di-GMP level, we predict that intracellular levels of this second messenger will be decreased in the presence of BSA+Ca^2+^. To test this hypothesis, we measured the intracellular c-di-GMP concentration in SS medium and SS supplemented with BSA+Ca^2+^.

**Fig 4:**
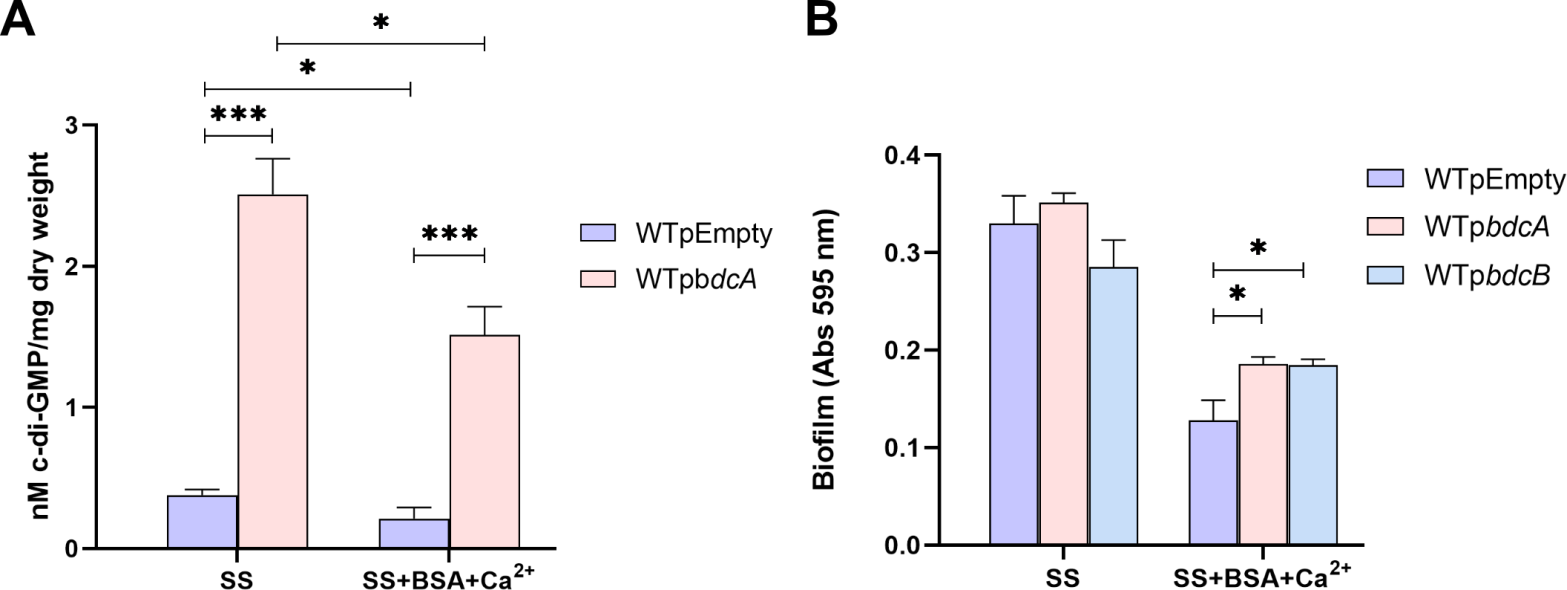
BSA+Ca^2+^-mediated decrease in c-di-GMP levels correlates with biofilm inhibition. (A) Intracellular c-di-GMP levels were measured in wild-type *B. bronchiseptica* or *Bb*p*bdcA* cells grown in SS or SS supplemented with 2.0 mg/mL BSA and 2.0 mM CaCl_2_. The results are the average of three independent experiments. * and *** Indicate significant differences (*P* < 0.05 and *P* < 0.01, respectively, by ANOVA). (B) Biofilm formation on PVC 96-well plates of overnight cultures of wild-type *B. bronchiseptica*, *Bb*pBdcA, and *Bb*pBdcB grown in SS or SS supplemented with 2.0 mg/mL BSA and 2.0 mM CaCl_2_. Biofilm was stained with CV and quantified after resuspension in 33% (vol/vol) acetic acid. Results are the average of at least three independent experiments. * indicates significant differences (*P* < 0.05. ANOVA).

Given that intracellular c-di-GMP levels might be low under unstimulated conditions, making a decrease harder to detect, we also evaluated c-di-GMP levels in a strain overexpressing an active DGC (BdcA). We previously showed that this strain produces higher levels of c-di-GMP (16). As shown in Fig. 4A, the addition of BSA+Ca^2+^ led to a significant decrease in intracellular c-di-GMP levels in both the WT and the strain expressing the DGC BdcA from a plasmid.

To test whether these changes in c-di-GMP levels correlate with biofilm formation, we evaluated the biofilm formation of strains expressing DGCs from a plasmid. Specifically, we expressed two different DGCs, BdcA and BdcB, which are membrane-localized and cytosolically localized DGCs, respectively (5, 17).

In the wild-type strain, the observed twofold decrease in intracellular c-di-GMP levels in the presence of BSA+Ca^2+^ (Fig. 4A) correlates with a similar twofold reduction in biofilm formation under the same conditions (Fig. 4B). This correlation supports the hypothesis that biofilm inhibition by BSA+Ca^2+^ is mediated through a reduction in c-di-GMP levels. Notably, biofilm formation was still observed in strains expressing a DGC from a plasmid (*BbpbdcA* and *BbpbdcB*), highlighting the role of elevated c-di-GMP levels in promoting biofilm formation even in the presence of BSA+Ca^2+^.

### Exploring PDEs and BvgR influence on biofilm formation

As shown above, c-di-GMP levels are diminished in the presence of BSA+Ca^2+^; thus, it is possible that one or more PDEs are activated upon the addition of these compounds. Therefore, if these PDEs are deleted from the *B. bronchiseptica* genome, biofilm inhibition should not occur upon the addition of BSA+Ca^2+^.

*B. bronchiseptica* has nine putative proteins with a PDE-associated EAL domain encoded in its genome. Among these, LapD, BB2109, and BvgR have degenerate EAL domains, suggesting they likely lack PDE activity. BB2957 and BB3317 are predicted to be dual-function proteins that may exhibit both DGC and PDE activities. Therefore, these proteins were excluded from our analysis. Among the remaining candidates, BB2110 (we propose the name Phosphodiesterase B, PdeB) is a bona fide EAL-containing protein. Multiple attempts to delete *bb2110* were unsuccessful. This narrowed our focus to three potential candidates: the two membrane-associated PDEs, PdeC and PdeD, and a cytosolic PDE, PdeA, previously described by our team (18). Although BvgR does not possess essential amino acids for PDE activity, we have described its importance in biofilm formation (19); therefore, we included this protein in the analysis.

**Fig 5:**
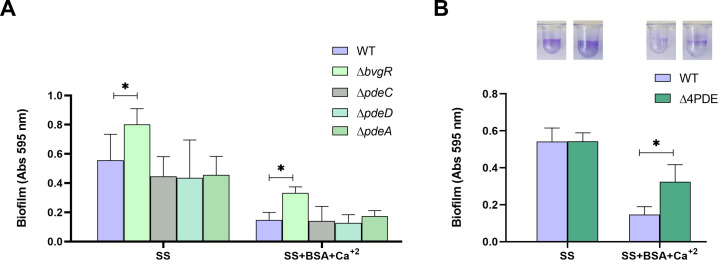
Exploring the influence of PDEs and BvgR on biofilm formation. Biofilm formation on PVC 96-well plates of overnight cultures of wild-type *B. bronchiseptica* and individual PDE mutants (**A**) or the *Bb*Δ4PDE mutant (**B**) grown in SS or SS supplemented with 2.0 mg/mL BSA and 2.0 mM CaCl_2_. Biofilm was stained with CV and quantified after resuspension in 33% (vol/vol) acetic acid. Results are the average of at least three independent experiments. * and ** indicate significant differences (*P* < 0.05 and *P* < 0.01, respectively, by ANOVA).

To test our hypothesis, we constructed individual mutations in the genes encoding each of these PDEs. All strains were evaluated for biofilm formation on a plastic surface using the CV assay. As previously described, the *Bb*Δ*bvgR* mutant strain formed more biofilm than the wild type in standard SS (Fig. 5A). Although the biofilm levels of the *pdeC* and *pdeD* mutants in SS appeared qualitatively lower than those of the wild type, no significant differences were observed. When grown in the presence of BSA+Ca^2+^, none of the PDE mutants showed significant differences compared to the wild-type strain (Fig. 5A). However, a modest but significant increase in biofilm formation was observed in the *Bb*Δ*bvgR* mutant in the presence of BSA+Ca^2+^ (Fig. 5A).

We also deleted all four genes coding for PDEs in case of potential redundancy. The quadruple mutant (*Bb*Δ4PDE) showed no difference from the WT in standard SS medium, but the *Bb*Δ4PDE showed significantly more biofilm formation than the wild-type strain in the presence of BSA+Ca^2+^ (Fig. 5B), consistent with our hypothesis above. Notably, the triple mutant (*Bb*Δ3PDE), which retains functional BvgR, slightly prevented the inhibitory effect of BSA+Ca^2+^ on biofilm formation (). Although the difference observed in the *Bb*Δ3PDE mutant is statistically significant, as evidenced by the CV-stained wells, it is not as pronounced as in the *Bb*Δ4PDE mutant. Nevertheless, these results suggest that the function of the three PDEs is important in this pathway. Taken together, BSA+Ca^2+^ likely stimulates the production and/or activity of two or more PDEs to reduce cellular levels of c-di-GMP.

### BSA + Ca^2+^ effect on BrtA surface levels and biofilm formation

**Fig 6:**
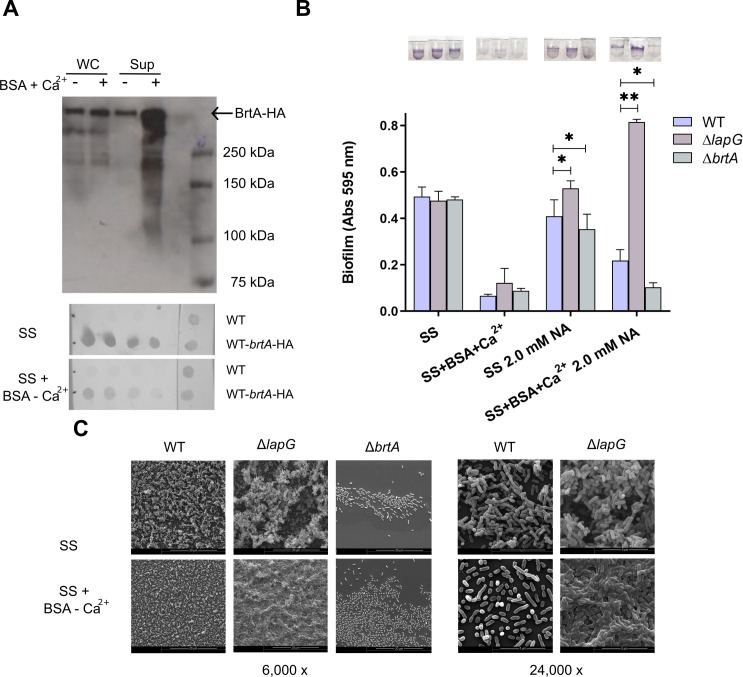
Effect of BSA+Ca^2+^ on BrtA secretion and biofilm formation. (A) Immunoblot analysis to determine BrtA level in whole-cell (WC) lysates and supernatants (by western blot) (top panel) or on the bacterial surface (by dot blot) (bottom panel) from late-phase growth culture of wild-type *B. bronchiseptica* grown in SS or SS supplemented with 2.0 mg/mL BSA and 2.0 mM CaCl_2_. The right blot in the dot blot shows Ponceau Red staining of the samples indicating cell biomass. (B) Biofilm formation on PVC 96-well plates of overnight cultures of wild-type *B. bronchiseptica*, *Bb*Δ*lapG,* and *BbΔbrtA* grown in SS or SS supplemented with 2.0 mg/mL BSA and 2.0 mM CaCl_2_ with the indicated concentrations of NA. Biofilm was stained with CV and quantified after resuspension in 33% (vol/vol) acetic acid. Results are the average of at least three independent experiments. * indicates significant differences (*P* < 0.05. ANOVA). (C) Scanning electron microscopy of 24 h cultures of *B. bronchiseptica* strains presented in panel B in the presence of 2.0 mM NA.

The effect of BSA + Ca^2+^ was observed in all virulent phases, but *B. bronchiseptica* biofilm formation peaks in the intermediate phase. Therefore, we decided to evaluate this phase for impacts on BrtA secretion and surface levels. Previously, we described BrtA as the adhesin responsible for biofilm formation in the intermediate phase (2.0 mM NA) and that the elaboration of this protein on the cell surface is regulated by c-di-GMP (6). Using a 2.0 mM concentration of NA, we assessed the effect of BSA+Ca^2+^ on BrtA secretion. The supernatant of wild-type bacteria in the presence of BSA+Ca^2+^ exhibited significantly higher levels of BrtA, but no difference in whole-cell levels (Fig. 6A, top panel). Moreover, the presence of BrtA on the bacterial surface, evidenced by a whole-cell dot blot, was consistent with the increased secreted BrtA phenotype (Fig. 6A, bottom panels).

The elevated BrtA in the supernatant and the absence of this protein on the cell surface when cells were treated with BSA+Ca^2+^ may explain the reduction in biofilm formation. If this hypothesis holds true, the inability to reduce BrtA levels on the surface would inhibit the effect of BSA+Ca^2+^. Deletion of the BrtA-specific protease LapG impedes BrtA release into the extracellular media (6). We evaluated the biofilm formation of wild type and *Bb*Δ*lapG* on PVC and glass surfaces (Fig. 6B and C). As expected, based on our hypothesis, the *lapG* mutant did not exhibit a reduced capacity for biofilm formation in the presence of BSA+Ca^2+^. Moreover, biofilm formation, measured by CV, was observed to be greater than that of the WT strain in the presence of 2.0 mM NA. To better understand this phenomenon, we examined biofilm formation using SEM. As shown in Fig. 6C, the *lapG* mutant shows a different phenotype under SS +BSA + Ca^2+^ at 2.0 mM NA, adhering more uniformly and failing to form the characteristic 3D structures observed in the WT.

## DISCUSSION

In our proposed model, the infection process of *B. bronchiseptica* begins with the colonization of the respiratory epithelium, where the bacteria may persist asymptomatically for extended periods. This persistence is facilitated by the formation of biofilm-like structures, which enable the bacteria to evade host immune responses and persist in the host. The bacteria express important adhesins, including FHA and BrtA, and secrete eDNA, contributing to biofilm persistence. The local concentration of albumin and calcium in this zone may not be sufficient to trigger a biofilm disruption response. Under unknown conditions, the bacteria can detach from the biofilm and progress toward the lower respiratory tract and lungs. In this environment, they are likely exposed to concentrations of albumin and calcium high enough to stimulate the secretion of ACT, potentially leading to biofilm inhibition. Together, this model raises the hypothesis that ACT secretion, driven by these factors, may contribute to biofilm inhibition in the lungs of infected animals. In addition, in this situation, c-di-GMP levels would be low with loss of cell-surface BrtA, consistent with reduced biofilm formation. We have previously reported that high levels of c-di-GMP inhibit Type III secretion system (TTSS) activity (18). Therefore, in the presence of albumin and calcium, *B. bronchiseptica* in the lung would also be expressing the TTSS system, necessary to counteract the host’s immune response.

The results presented here support the model proposed above. The presence of albumin (mimicked by BSA in our experiments) and calcium induced an increase in extracellular ACT levels and a loss of cell-surface BrtA. The inability to produce extracellular ACT or release cell-surface BrtA partially prevented the inhibitory effect of BSA+Ca^2+^, demonstrating that this inhibition is mediated by multiple factors, including ACT, BrtA, and potentially other unidentified factors. Although FHA is an important adhesin for biofilm formation, likely facilitating FHA-FHA bridges between bacteria, our experiments did not show that its presence affects the action of BSA+Ca^2+^. However, when FHA’s release was blocked through the deletion of the *degP* gene, we observed that *B. bronchiseptica* could not properly form biofilm. This finding is particularly intriguing, as other studies have shown that exogenous FHA inhibits biofilm formation in the closely related *B. pertussis* (10).

Our findings also confirm the involvement of c-di-GMP in biofilm regulation by albumin and calcium. Inhibition by calcium of purified PDEs has been described previously (20, 21). However, experiments with whole bacteria have revealed variable outcomes, depending on the experimental setup and bacterial species. For instance, for *Vibrio fischeri*, calcium has been reported to either increase or decrease intracellular c-di-GMP levels, depending on the growth media used (22, 23). In this study, we observed a reduction in c-di-GMP levels in the presence of BSA and calcium. However, we were unable to establish a direct link between ACT levels and c-di-GMP, suggesting that albumin and calcium may independently impact ACT and BrtA. Previously, we reported that *B. bronchiseptica* overexpressing the DGC BdcA exhibited lower total ACT levels (18), indicating that a possible connection between ACT and BrtA secretion cannot be ruled out.

The second messenger c-di-GMP regulates various phenotypes through the activation of distinct PDEs and DGCs, each responsive to different signals, as recently proposed in the Bowtie model (24). In this scenario, the phenotype would only be observed when the specific PDE is removed. In our study, we found that three PDEs (PdeA, PdeC, and PdeD) are important in the response to BSA+Ca^2+^ since the strain *Bb*Δ3PDE decreased biofilm formation less than the WT when exposed to these components. However, the differences were not as pronounced as those observed with the Δ4PDE, which includes a deletion in the BvgR gene, as suggested by the CV staining. Interestingly, there is no evidence to support that BvgR is an active PDE (19). Further studies are needed to identify other factors involved in this regulation and to understand how the bacteria sense the presence of albumin and calcium.

Our findings may be relevant to aspects of infection. For example, there is evidence that high concentrations of albumin and calcium resemble the environment in the host’s nostrils (12). Infiltration of immune cells from nasal tissue to the site of infection transports significant amounts of albumin and calcium, which are present in the extracellular fluid. *Bordetella spp*., established as microcolonies on the epithelial surface, may sense this as a signal to disassemble biofilm or prevent the formation of new biofilm structures, facilitating its spread to other regions where the immune response has not yet developed.

Finally, we would like to emphasize an important consideration regarding the evidence that albumin and calcium inhibit biofilm formation *in vitro*. This inhibition occurs under conditions with high concentrations of albumin and calcium, whereas typical media used in *in vitro* experiments contain significantly lower levels of both, perhaps explaining why these phenotypes have not been observed previously.

## MATERIALS AND METHODS

### Bacterial strains and growth conditions

*B. bronchiseptica* strains () were grown at 37°C either on Bordet-Gengou agar (BGA, Difco) supplemented with 10% (vol/vol) defibrinated sheep blood or in modified Stainer-Scholte medium (SS) at 160 rpm or statically, respectively. Media were supplemented with gentamicin (50 µg/mL) or streptomycin (200 µg/mL) as needed. Bacterial strains and plasmids used in this work are listed in .

*Escherichia coli* (DH5α and S17-1) strains were cultured with lysogeny broth (LB) either in test tubes or on LB agar plates (1.5% agar). Gentamicin was added to the medium at 10 µg/mL final concentration when appropriate. Replicative plasmids were introduced to *E. coli* by electroporation using standard techniques. Non- and replicating plasmids were introduced into *B. bronchiseptica* by conjugation. The yeast strain InvSc1 (*Saccharomyces cerevisiae*; Invitrogen) was routinely cultured on a YPD medium. To select plasmids carrying the URA3 gene, yeast was grown on a YNB medium with a complete supplemental mixture minus uracil.

### Strain and plasmid construction

All oligonucleotide primers used in the study are listed in . Cloning was performed by *in vivo* recombination in yeast, as described by Shanks (25). Briefly, a plasmid derived from the pMQ30 allelic replacement vector was utilized to create knockout strains. Two homologous DNA regions flanking the target genomic area were amplified by PCR, designated F1 and F2, using primers with over 30 additional bases to aid in recombination with adjacent fragments. These fragments were inserted into the pMQ30 plasmid using yeast cloning methods, resulting in pMQ30F1F2. The plasmid was then recovered from yeast and electroporated into *E. coli* S17-1. All constructs were confirmed by PCR and DNA sequencing. Allelic replacement mutants were generated as previously described (26). In brief, the plasmid was introduced into wild-type *B. bronchiseptica* via biparental conjugation. Simple recombinants were selected using streptomycin and gentamicin. For a detailed protocol of *B. bronchiseptica* conjugation, see reference (26). Clones resistant to both antibiotics were then grown in SS medium without antibiotics and plated on LB containing 11% sucrose to isolate clones that had lost the plasmid. These clones were checked for gentamicin sensitivity, and the deletion was confirmed by PCR using primers that flank the deleted region.

### Biofilm formation assays

*B. bronchiseptica* biofilm assays were performed as previously reported by our group (5). Cultures with an OD_650nm_ of 0.1 were prepared by resuspending colonies grown on SS (1.5% [wt/vol] agar) supplemented with 10% (vol/vol) defibrinated sheep blood into SS. One hundred microliter of these cultures was then pipetted into a 96-well U-bottom microtiter plate (polyvinylchloride, PVC) and incubated statically at 37°C for 24 hours. After incubation, planktonic bacteria were removed by washing, and attached bacteria were stained with a 0.1% wt/vol CV solution. The stain was then solubilized in 120 µL of a 33% (vol/vol) acetic acid solution. Biofilm formation was quantified by measuring Abs at 595 nm of 100 µL of the solubilized stain solution. Nicotinic acid was added at specified concentrations when used, and bovine serum albumin (BSA) and CaCl_2_ were added where indicated, at final concentrations of 2 mg/mL and 2.0 mM, respectively. The experiments were repeated at least three times, each with four technical replicates.

### Measurements of c-di-GMP levels

C-di-GMP levels were analyzed via LC-MS as previously described (5). Briefly, four replicates of each strain and condition were harvested and resuspended in 250 µL of extraction buffer (methanol-acetonitrile-water [40:40:20] plus 0.1 N formic acid at −20°C) and incubated at −20°C for 30 min. The cell debris was pelleted for 5 min at 4°C, and the supernatant containing the nucleotide extract was immediately adjusted to a pH of ~7.5 with 15% (NH_4_)_2_HCO_3_ and stored on dry ice prior to analysis. The resultant extract was analyzed via LC-MS using the LC-20AD high-performance LC system (Shimadzu, Columbia, MD) coupled to a Finnigan TSQ Quantum Discovery MAX triple-quadrupole mass spectrometer (Thermo Electron Corp., San Jose, CA).

### Western blots

*B. bronchiseptica* strains were grown for 48 hours on BGA plates and then transferred to SS liquid culture for overnight growth. Samples were collected, and the OD_650nm_ was standardized. After centrifugation at 13,500 rpm for 3 minutes, the supernatants intended for HA-tag detection were filtered through a low-binding protein 0.22 µm filter, transferred to new tubes, and concentrated using an Amicon Ultra centrifugal filter with a molecular weight cutoff (MWCO) of 10.0 kDa. Bacterial pellets were resuspended in PBS to achieve an OD_650nm_ of 10 for BrtA-HA detection.

The quantity of protein in the supernatants was measured with the BCA Pierce Kit. All samples were boiled for 10 min and centrifuged before loading. Proteins were separated by SDS-PAGE 8% and transferred to a nitrocellulose membrane. BrtA-HA was detected with anti-HA diluted 1:2,000 in 3% wt/vol BSA in TBS overnight at 4°C, followed by incubation with anti-mouse IgG conjugated to horseradish peroxidase (HRP) (1:15,000) (Invitrogen) in TBS-0.01% (vol/vol) Tween containing 3% (wt/vol) BSA at room temperature for 2.5 h. Detection was performed using Clarity Western ECL Substrate (Bio-Rad #1705060) according to the manufacturer’s instructions.

### Dot blot

*B. bronchiseptica* strains were grown as described above for western blot analysis. An aliquot of 1.5 mL was centrifuged at 16,000 × *g* for 3 min and the pellets were washed two times with 1 mL of buffer (Tris 20 mM, MgCl_2_ 10 mM, pH = 8.0) and finally resuspended in the same buffer to obtain a bacterial suspension with an OD of 1. The bacterial suspensions were diluted serially twofold (½, ¼, ⅛) and 10 µL of these samples were pipetted onto a nitrocellulose membrane, or pipetted directly in triplicate. Once dried, the membrane was incubated for 1 h with a blocking buffer (5% (wt/vol) nonfat milk in PBS 0,01% (vol/vol) Tween 20). BrtA and FHA were detected using a mouse monoclonal anti-HA or anti-FHA antibody (1:5,000 in blocking buffer) as the primary antibody and a goat anti-mouse or anti-rabbit monoclonal antibody, respectively, conjugated to HRP as the secondary antibody. Non-blocked samples were stained with Ponceau red to confirm equal protein concentration of samples.

### Scanning electron microscopy

Biofilm assays for scanning electron microscopy were conducted as previously described by our group (5). *B. bronchiseptica* was cultured in SS medium on glass coverslips, which were vertically submerged in plastic tubes for 24 hours. The coverslips were then subjected to a CO_2_ critical-point procedure using an EmiTech K850 and sputter-coated with gold. Samples were examined with a scanning electron microscope (FEI Quanta 200), and images were processed using the Image Soft Imaging System ADDA II. At least two independent samples were analyzed per strain, with a majority of each sample scanned and representative images selected for processing.

### Statistical analysis

Each experiment included at least two biological replicates (as indicated in each experiment). Data were analyzed for statistical significance using a one-way ANOVA followed by Tukey’s multiple-comparison test to assess differences among groups. The significance level is specified in the figure legend of each experiment.

## Data Availability

The GenBank accession number for the *B. bronchiseptica* RB50 genome is NC_002927.3. The gene identification number and locus tag, respectively, for *bdcB* are 2661408 and BB_RS19575, *pdeA* are 2660213 and BB_RS13380, *pdeC* are 2661280 and BB_RS15655 and *pdeD* are 2661612 and BB_RS15715. The supporting information available at http://sedici.unlp.edu.ar/handle/10915/177239.

## References

[1] Harvill ET, Cotter PA, Miller JF. 1999. Pregenomic comparative analysis between Bordetella bronchiseptica RB50 and Bordetella pertussis tohama I in murine models of respiratory tract infection. Infect Immun 67:6109–6118. doi:10.1128/IAI.67.11.6109-6118.1999 10531274 PMC97000

[2] Nicholson TL, Brockmeier SL, Sukumar N, Paharik AE, Lister JL, Horswill AR, Kehrli ME, Loving CL, Shore SM, Deora R. 2017. The Bordetella Bps polysaccharide is required for biofilm formation and enhances survival in the lower respiratory tract of swine. Infect Immun 85:e00261-17. doi:10.1128/IAI.00261-17 28559403 PMC5520422

[3] Gueirard P, Ave P, Balazuc A-M, Thiberge S, Huerre M, Milon G, Guiso N. 2003. Bordetella bronchiseptica persists in the nasal cavities of mice and triggers early delivery of dendritic cells in the lymph nodes draining the lower and upper respiratory tract. Infect Immun 71:4137–4143. doi:10.1128/IAI.71.7.4137-4143.2003 12819105 PMC162036

[4] Irie Y, Mattoo S, Yuk MH. 2004. The Bvg virulence control system regulates biofilm formation in Bordetella bronchiseptica. J Bacteriol 186:5692–5698. doi:10.1128/JB.186.17.5692-5698.2004 15317773 PMC516841

[5] Sisti F, Ha D-G, O’Toole GA, Hozbor D, Fernández J. 2013. Cyclic-di-GMP signalling regulates motility and biofilm formation in Bordetella bronchiseptica*.* Microbiology (Reading) 159:869–879. doi:10.1099/mic.0.064345-0 23475948 PMC4085988

[6] Ambrosis N, Boyd CD, O´Toole GA, Fernández J, Sisti F. 2016. Homologs of the LapD-LapG c-di-GMP effector system control biofilm formation by Bordetella bronchiseptica. PLoS ONE 11:e0158752. doi:10.1371/journal.pone.0158752 27380521 PMC4933386

[7] Sloan GP, Love CF, Sukumar N, Mishra M, Deora R. 2007. The Bordetella Bps polysaccharide is critical for biofilm development in the mouse respiratory tract. J Bacteriol 189:8270–8276. doi:10.1128/JB.00785-07 17586629 PMC2168688

[8] Conover MS, Sloan GP, Love CF, Sukumar N, Deora R. 2010. The Bps polysaccharide of Bordetella pertussis promotes colonization and biofilm formation in the nose by functioning as an adhesin. Mol Microbiol 77:1439–1455. doi:10.1111/j.1365-2958.2010.07297.x 20633227 PMC2939936

[9] Cattelan N, Dubey P, Arnal L, Yantorno OM, Deora R. 2016. Bordetella biofilms: a lifestyle leading to persistent infections. Pathog Dis 74:ftv108. doi:10.1093/femspd/ftv108 26586694 PMC4830220

[10] Serra DO, Conover MS, Arnal L, Sloan GP, Rodriguez ME, Yantorno OM, Deora R. 2011. FHA-mediated cell-substrate and cell-cell adhesions are critical for Bordetella pertussis biofilm formation on abiotic surfaces and in the mouse nose and the trachea. PLoS One 6:e28811. doi:10.1371/journal.pone.0028811 22216115 PMC3245231

[11] Hoffman C, Eby J, Gray M, Heath Damron F, Melvin J, Cotter P, Hewlett E. 2017. Bordetella adenylate cyclase toxin interacts with filamentous haemagglutinin to inhibit biofilm formation in vitro. Mol Microbiol 103:214–228. doi:10.1111/mmi.13551 27731909 PMC5218874

[12] Gonyar LA, Gray MC, Christianson GJ, Mehrad B, Hewlett EL. 2017. Albumin, in the presence of calcium, elicits a massive increase in extracellular bordetella adenylate cyclase toxin. Infect Immun 85:e00198-17. doi:10.1128/IAI.00198-17 28396321 PMC5442642

[13] Bumba L, Masin J, Macek P, Wald T, Motlova L, Bibova I, Klimova N, Bednarova L, Veverka V, Kachala M, Svergun DI, Barinka C, Sebo P. 2016. Calcium-driven folding of RTX domain β-rolls ratchets translocation of RTX proteins through type I secretion ducts. Mol Cell 62:47–62. doi:10.1016/j.molcel.2016.03.018 27058787

[14] Johnson RM, Nash ZM, Dedloff MR, Shook JC, Cotter PA. 2021. DegP initiates regulated processing of filamentous hemagglutinin in Bordetella bronchiseptica. MBio 12:e0146521. doi:10.1128/mBio.01465-21 34182780 PMC8263021

[15] Nash ZM, Inatsuka CS, Cotter PA, Johnson RM. 2024. Bordetella filamentous hemagglutinin and adenylate cyclase toxin interactions on the bacterial surface are consistent with FhaB-mediated delivery of ACT to phagocytic cells. MBio 15:e0063224. doi:10.1128/mbio.00632-24 38534159 PMC11077949

[16] Belhart Keila, Gutierrez M de la P, Zacca F, Ambrosis N, Cartelle Gestal M, Taylor D, Dahlstrom KM, Harvill ET, O’Toole GA, Sisti F, Fernández J. 2019. Bordetella bronchiseptica diguanylate cyclase BdcA regulates motility and is important for the establishment of respiratory infection in mice. J Bacteriol 201:e00011-19. doi:10.1128/JB.00011-19 31209073 PMC6689298

[17] Belhart K, Sisti F, Gestal MC, Fernández J. 2023. Bordetella bronchiseptica diguanylate cyclase BdcB inhibits the type three secretion system and impacts the immune response. Sci Rep 13:7157. doi:10.1038/s41598-023-34106-x 37130958 PMC10154355

[18] Gutierrez María de la Paz, Wong TY, Damron FH, Fernández J, Sisti F. 2022. Cyclic di-GMP Regulates the Type III Secretion System and virulence in Bordetella bronchiseptica. Infect Immun 90:e0010722. doi:10.1128/iai.00107-22 35612302 PMC9202433

[19] Gutierrez Maria de la Paz, Damron FH, Sisti F, Fernández J. 2024. BvgR is important for virulence-related phenotypes in Bordetella bronchiseptica Microbiol Spectr 12:e0079424. doi:10.1128/spectrum.00794-24 39365045 PMC11537037

[20] Ross P, Weinhouse H, Aloni Y, Michaeli D, Weinberger-Ohana P, Mayer R, Braun S, de Vroom E, van der Marel GA, van Boom JH, Benziman M. 1987. Regulation of cellulose synthesis in Acetobacter xylinum by cyclic diguanylic acid. Nature New Biol 325:279–281. doi:10.1038/325279a0 18990795

[21] Tamayo R, Tischler AD, Camilli A. 2005. The EAL domain protein VieA is a cyclic diguanylate phosphodiesterase. J Biol Chem 280:33324–33330. doi:10.1074/jbc.M506500200 16081414 PMC2776828

[22] Tischler AH, Vanek ME, Peterson N, Visick KL. 2021. Calcium-responsive diguanylate cyclase CasA drives cellulose-dependent biofilm formation and inhibits motility in Vibrio fischeri. MBio 12:e0257321. doi:10.1128/mBio.02573-21 34749532 PMC8576532

[23] Shrestha P, Razvi A, Fung BL, Eichinger SJ, Visick KL. 2022. Mutational analysis of Vibrio fischeri c-di-GMP-modulating genes reveals complex regulation of motility. J Bacteriol 204:e0010922. doi:10.1128/jb.00109-22 35758751 PMC9295575

[24] Vasenina A, Fu Y, O’Toole GA, Mucha PJ. 2024. Local control: a hub-based model for the c-di-GMP network. mSphere 9:e0017824. doi:10.1128/msphere.00178-24 38591888 PMC11237430

[25] Shanks RMQ, Caiazza NC, Hinsa SM, Toutain CM, O’Toole GA. 2006. Saccharomyces cerevisiae-based molecular tool kit for manipulation of genes from gram-negative bacteria. Appl Environ Microbiol 72:5027–5036. doi:10.1128/AEM.00682-06 16820502 PMC1489352

[26] Ambrosis N, Fernández J, Sisti F. 2020. Counter-selection method for markerless allelic exchange in Bordetella bronchiseptica based on sacB gene from Bacillus subtilis. Curr Protoc Microbiol 59:e125. doi:10.1002/cpmc.125 33166051

